# Microbial Contamination of Gym Equipment: Diversity Patterns, Temporal Dynamics, Staphylococcus Hotspots, and Device-Level Risk Indices

**DOI:** 10.3390/pathogens15070707

**Published:** 2026-07-06

**Authors:** Alexander Martens, Markus Schauer, Mohamad Motevalli, Susanne Mair, Brigitte König

**Affiliations:** 1 Institute for Medical Microbiology and Virology, University of Leipzig Medical Center, University of Leipzig, 04103 Leipzig, Germany; 2 Department of Sport Science, German University of Health & Sport (DHGS), 85737 Ismaning, Germany; markus.schauer@dhgs-hochschule.de (M.S.); moha.motevalli@dhgs-hochschule.de (M.M.); susanne.mair.dz@edu.dhgs-hochschule.de (S.M.)

**Keywords:** microbial pathogens, surface microbiome, contact transmission, *Staphylococcus aureus*, skin microbiota, microbial diversity, bacterial interactions, environmental microbiology, athletic environments, fitness club

## Abstract

Background: Public fitness facilities are high-contact environments that facilitate microbial transfer via shared surfaces; however, temporal dynamics and device-specific contamination patterns remain insufficiently characterized. Methods: A repeated-measures observational study was conducted in a fitness facility over five consecutive weekdays (Monday to Friday). A total of 180 surface samples were collected from 12 gym devices, each sampled three times daily (morning, noon, and evening). Surface-associated cultivable bacteria were recovered using culture-based methods followed by MALDI-TOF MS identification. Ecological metrics, including species richness and Shannon diversity, were calculated, and taxa were classified by origin (skin-associated versus environmental). Device-specific contamination profiles were developed using a composite index incorporating pathogen presence, contamination frequency, and persistence. Temporal trends and predictors of contamination were analyzed using mixed-effects regression models. All statistical analyses were performed in R. Results: A total of 248 bacterial isolates were identified, representing 61 species across 32 families, with a predominance of skin-associated taxa (72.2%). Sampling time point was a strong independent predictor of contamination (adjusted OR for noon vs. morning: 7.19; *p* < 0.001). While overall microbial diversity remained stable across devices (Shannon index, *p* = 0.44), substantial heterogeneity was observed in pathogen prevalence, multispecies burden, and persistence. The functional trainer and leg extension showed the highest composite risk scores (42.3%), while the ab crunch machine and upper body ergometer demonstrated significantly increasing contamination trends over the sampling period (*p* < 0.05). Co-occurrence analysis showed nonrandom microbial associations, with the strongest positive links between *Micrococcus luteus* and *Staphylococcus saprophyticus* (Φ = 0.76) and *Staphylococcus aureus* (Φ = 0.61). Conclusions: Gym equipment surfaces harbor predominantly human-associated microbial communities exhibiting dynamic temporal contamination patterns, and on selected devices, increasing the baseline contamination across consecutive cleaning cycles. The findings indicate that contamination patterns on shared fitness equipment are dominated by taxa commonly associated with human skin and support targeted hygiene interventions focused on frequently contacted devices and periods of elevated contamination.

## 1. Introduction

Public fitness facilities represent dynamic, high-contact environments where repeated human interaction with shared surfaces creates favorable conditions for microbial transfer and persistence [1,2]. Frequently touched fomites such as equipment handles and handrails can act as reservoirs for microorganisms originating from human skin, respiratory secretions, and environmental sources, facilitating indirect transmission pathways [1,3,4]. Despite the implementation of routine cleaning and disinfection protocols, several studies have demonstrated that microorganisms can persist on inanimate surfaces for extended periods, particularly under conditions of repeated recontamination and variable hygiene compliance [5,6].

The microbiology of built environments is strongly influenced by human occupancy, with indoor surface communities typically dominated by skin-associated taxa such as *Staphylococcus* and *Micrococcus* species [7,8,9]. These organisms are readily transferred via direct contact and can survive on abiotic surfaces depending on environmental conditions and material properties [6,10]. While many are commensal, clinically relevant species, including *Staphylococcus aureus* (*S. aureus*), are of particular concern due to their well-established role in skin and soft tissue infections and their capacity for transmission via shared surfaces [11,12]. This highlights the importance of not only characterizing microbial presence, but also taxonomic composition in high-contact public environments.

Investigations of microbial contamination in fitness centers and similar communal environments have typically relied on narrow sampling frameworks, often limited to a small subset of surfaces or single time points [1,13,14,15]. Such approaches may inadequately capture the temporal dynamics of microbial accumulation driven by fluctuations in user density, diurnal activity patterns, and cleaning interventions. Evidence from indoor microbiome studies suggests that microbial load and composition can change substantially over short time scales in response to human activity [16,17], highlighting the need for repeated-measures designs to accurately assess exposure risk. Additionally, variation in equipment design, such as differences in material composition, surface geometry, and contact frequency, may create device-specific ecological niches that remain poorly characterized.

Beyond overall contamination burden, the structure and diversity of microbial communities provide critical insight into contamination sources and potential health implications [1,16,18]. Quantitative metrics such as species richness and diversity indices enable the assessment of ecological complexity [19], while the classification of taxa by origin (e.g., skin-associated versus environmental) helps elucidate transmission pathways [20,21]. Furthermore, the detection of opportunistic pathogens, patterns of multi-species co-contamination, and interspecies associations may reflect underlying ecological interactions that influence microbial persistence and resilience on surfaces [22,23,24]. Although previous studies have documented microbial contamination in gym environments [1,2,3,4,13,14,15], most have focused primarily on overall bacterial burden or the detection of selected pathogens, with comparatively limited attention to integrated ecological characterization across multiple equipment types and repeated temporal sampling intervals. Consequently, temporal variability, device-specific contamination patterns, microbial community structure, and co-contamination dynamics within real-world operational fitness settings remain insufficiently characterized.

To address these gaps, the present study employs a repeated-measures observational design to systematically characterize cultivable bacterial contamination patterns across a diverse set of gym equipment in a real-world fitness facility. By combining standardized surface sampling, culture-based identification, and quantitative ecological metrics, this study aims to (i) delineate the taxonomic composition and ecological origin of cultivable bacterial populations on high-contact gym surfaces, (ii) evaluate temporal dynamics in contamination across intra-day and inter-day cycles, and (iii) develop device-specific contamination profiles incorporating pathogen presence, persistence, and multi-species burden. The novelty of this study lies in its integrated analytical framework, combining repeated temporal sampling with device-level ecological profiling to provide a multidimensional assessment of cultivable bacterial contamination dynamics in fitness environments. Specifically, the study simultaneously evaluates microbial diversity, contamination persistence, ecological origin, and co-occurrence patterns across multiple equipment categories under routine operational conditions. While culture-based methods may not capture the full microbial diversity present on gym surfaces, they enable the characterization of temporal and device-specific patterns within the cultivable bacterial fraction. This integrated approach provides a more granular understanding of microbial exposure risks in fitness environments and may inform targeted hygiene and infection prevention strategies.

Accordingly, the present study advances previous gym contamination surveys by integrating repeated temporal sampling with device-specific ecological profiling, enabling the simultaneous assessment of microbial diversity, ecological origin, contamination persistence, and co-occurrence patterns across multiple equipment types under routine operational conditions.

## 2. Materials and Methods

### 2.1. Study Design and Setting

This study was designed as a repeated-measures observational investigation of cultivable bacterial contamination patterns on gym equipment surfaces. The study was conducted in accordance with the Declaration of Helsinki and applicable national regulations. Sampling was conducted in a fitness studio in Munich, Germany, over five consecutive weekdays (Monday through Friday). The study design incorporated structured repeated sampling to capture fluctuations driven by human usage patterns and routine cleaning practices. As a descriptive and exploratory study conducted within a fitness facility, the objective was to characterize temporal and device-specific contamination patterns under real-world operating conditions and obtain high-resolution contamination data within a standardized operational environment, rather than to comprehensively assess the total microbial community, perform a formal microbial risk assessment, or generate broadly generalizable estimates across fitness facilities.

The facility followed routine daily cleaning procedures, typically conducted outside of operating hours, with particular attention to high-contact surfaces. Specifically, the gym employed a standardized disinfection protocol consisting of a quaternary ammonium-based surface disinfectant applied once daily after closing. Staff used pre-moistened disinfectant wipes to clean all equipment handles, touchscreen interfaces, and bench surfaces, ensuring full wetting of the contact area and a minimum manufacturer-recommended contact time of approximately 2–3 min. These procedures aligned with the national hygiene recommendations for the routine disinfection of high-contact surfaces in fitness facilities. No intermediate cleaning was performed during operating hours unless visibly soiled surfaces required ad hoc wiping by staff or users.

### 2.2. Sampling Sites and Timeline

A total of 12 training devices were selected for microbiological monitoring to represent a heterogeneous range of equipment types, including resistance machines, cable-based systems, and cardiovascular devices (Table 1). Selection was guided by expected variation in user interaction frequency, surface material composition, and handle geometry, all of which influence microbial transfer and persistence. Sampling was performed on device handles, which constitute the primary interface for direct hand contact and are therefore the most relevant surfaces for microbial transmission. For each device, a predefined handle region was consistently targeted across all sampling events. These included pulley grips, stabilization handles, rowing attachments, rotary cranks, and treadmill handrails.

Sampling was conducted three times daily at standardized time points: in the morning (prior to facility opening), at noon (12:00), and in the evening (immediately before closing). Morning samples were collected following routine overnight cleaning procedures and thus represented a post-disinfection baseline. Because the facility performed only one scheduled disinfection event per day, noon and evening samples reflected progressive microbial accumulation on surfaces that had not been re-cleaned since the previous night. Noon sampling captured microbial accumulation during peak daytime usage, while evening sampling reflected cumulative contamination after prolonged user exposure. This structured sampling framework resulted in 15 samples per device over the five-day study period and a total of 180 environmental samples.

### 2.3. Environmental Sampling Procedure

Environmental sampling was performed using sterile ESwab™ collection systems (Copan Italia S.p.A., Brescia, Italy) containing liquid Amies transport medium. Prior to sampling, the swab tip was moistened with the transport medium to enhance microbial recovery. Each predefined handle surface was then systematically swabbed using a standardized technique involving uniform pressure and multidirectional strokes to maximize coverage of the entire contact area. Following collection, all samples were transported to the laboratory and processed within a standardized time frame to preserve bacterial viability. Swabs were maintained in their transport medium at ambient temperature and processed within a short interval after collection, thereby minimizing potential alterations in microbial composition due to prolonged storage or environmental exposure. To ensure comparability across devices and sampling time points, the sampled surface area was standardized for all measurements. For each device, the entire accessible handle surface corresponding to the primary hand-contact region was systematically swabbed. Swabbing was performed using a uniform multidirectional technique with consistent pressure and coverage to minimize variability in microbial recovery attributable to operator-dependent factors.

### 2.4. Microbiological Culture and Isolation

From each ESwab sample, a 10 µL aliquot of transport medium was inoculated onto multiple culture media to facilitate broad-spectrum bacterial recovery and the selective isolation of clinically relevant groups. The primary media included Müller–Hinton agar supplemented with 5% sheep blood for general bacterial growth, mannitol salt agar for selective isolation of staphylococci, and Endo agar for the detection of Gram-negative organisms. To account for microbial heterogeneity within samples, colonies were selected based on distinct morphological characteristics, including differences in size, shape, color, and texture. Where multiple morphotypes were observed on a single plate, representative colonies of each type were chosen to capture within-sample diversity. This approach was applied consistently across all samples to minimize selection bias and ensure the inclusion of both dominant and less abundant organisms in downstream analyses.

All inoculated plates were incubated aerobically at 37 °C and examined after approximately 24 h. Plates exhibiting low or delayed growth were re-examined following extended incubation to allow for the detection of slower-growing organisms. After incubation, morphologically distinct colonies were selected as representative isolates and sub-cultured onto fresh media to obtain pure cultures prior to identification. To ensure data integrity and minimize the risk of external contamination, standard microbiological quality control procedures were implemented throughout the study. Culture media sterility was routinely verified prior to use, and all sample handling was performed using aseptic techniques. Negative controls, including unused swabs exposed to the sampling environment, were periodically incorporated to monitor for potential environmental or laboratory contamination. All procedures were performed in accordance with established microbiological standards and widely accepted guidelines for culture-based isolation and characterization [25].

### 2.5. Bacterial Identification and Preservation of Isolates

A sample was classified as positive for bacterial contamination if at least one viable bacterial isolate was detected and successfully identified following culture and analysis. This binary definition was applied consistently across all statistical analyses. Bacterial identification was performed using matrix-assisted laser desorption/ionization time-of-flight mass spectrometry (MALDI-TOF MS), enabling rapid and accurate taxonomic assignment based on protein spectral profiles. Spectral data were analyzed against a validated reference database, and identifications were interpreted according to established manufacturer-defined score thresholds. Species-level identification was accepted when spectral scores met the validated cut-off values, while lower scores were assigned at the genus level where appropriate. Presumptive *S. aureus* isolates underwent additional confirmatory testing using latex agglutination assays for coagulase detection to ensure diagnostic accuracy. Gram-negative isolates recovered on Endo agar were likewise identified using MALDI-TOF MS following subculture.

All confirmed bacterial isolates were stored at −80 °C in cryobank tubes containing an appropriate cryoprotective medium for long-term preservation, allowing for subsequent molecular or phenotypic characterization and maintained isolate availability for future analyses.

### 2.6. Microbial Outcomes and Analytical Variables

Microbial contamination was defined as the detection of at least one culturable bacterial isolate in each sample. Species richness was calculated as the number of distinct bacterial species identified per sample, while family richness reflected the number of unique bacterial families detected. Community diversity was quantified using the Shannon diversity index, which accounts for both species’ richness and evenness. When multiple isolates of the same species were recovered from a single sample, they were counted only once for the calculation of species and family richness; however, all isolates were retained for Shannon diversity index calculations, where they contributed as abundance-weighted observations.

Each isolate was classified according to ecological origin as either skin-associated or environmental based on established microbiological criteria. This classification was informed by established microbiological literature and reference database annotations [26,27,28] and reflects the predominant ecological associations reported for each taxon. The classification was used to calculate the ratio of environmental to skin-associated bacteria, reflecting shifts in microbial source contribution over time. It should be noted that several taxa may occur across multiple environments [17], and this categorization was applied as a pragmatic framework for the ecological interpretation of cultured isolates.

Staphylococcal contamination was evaluated at two levels: detection of any *Staphylococcus* species and specific identification of *S. aureus*. Multi-species contamination was defined as the presence of more than one bacterial species within a single sample. Temporal persistence was calculated as the proportion of consecutive sampling intervals in which at least one bacterial species was detected for a given device. A composite device-level risk score was defined a *priori* as a weighted index incorporating four parameters: *S. aureus* positivity (35%), presence of any *Staphylococcus* species (25%), overall bacterial detection rate (20%), and multi-species contamination frequency (20%). The weighting scheme was not derived from a validated risk model but was established *a priori* as an exploratory framework. Weighting was determined conceptually based on the relative epidemiological relevance of each parameter, with greater emphasis assigned to *S. aureus* due to its established association with fomite-mediated transmission in shared indoor environments [10,11,12]. The intermediate weighting assigned to overall *Staphylococcus* detection reflects the predominance, persistence, and frequent transfer of skin-associated staphylococci on high-contact surfaces in fitness environments [2,3,4], whereas overall bacterial detection rate and multi-species contamination were assigned equal weights as complementary indicators of microbial burden and ecological complexity rather than direct measures of infection risk. Accordingly, the composite index was intended as an exploratory tool to integrate multiple dimensions of contamination into a single device-level comparative metric.

### 2.7. Statistical Analysis

All statistical analyses were performed in R (version 4.5.1) using established packages for data management, descriptive statistics, comparative analyses, regression modeling, and visual illustrations. Results were presented using tables alongside complex heatmap, stacked bar charts, forest plot, violin plot, spaghetti plot, line plot, and correlation heatmap visualizations. Microbial diversity was quantified using species richness, family richness, and the Shannon diversity index, with device-level differences in Shannon diversity assessed using the Kruskal–Wallis test. Temporal changes in the environmental-to-skin bacterial ratio across the five-day sampling period were assessed using the Friedman test, followed by post hoc pairwise Wilcoxon signed-rank tests. Effect sizes for Friedman tests were expressed as Kendall’s W. *p*-values from multiple pairwise comparisons were adjusted using the Benjamini–Hochberg false discovery rate procedure. Species co-occurrence patterns were quantified using Phi correlation coefficients derived from 2 × 2 contingency tables, with statistical significance assessed by Fisher’s exact test. Temporal trends in device-specific metrics over the five-day period were evaluated using linear regression models with time treated as a continuous variable. Associations between predictors and bacterial outcomes, including *Staphylococcus* presence and overall contamination, were evaluated using mixed-effects logistic regression models. Fixed effects included time of day, weekday, and device type, while a random intercept for device was included to account for repeated measurements. Adjusted odds ratios with 95% confidence intervals were reported, and overall predictor significance was assessed using Type III likelihood ratio tests. Model discrimination was evaluated using the area under the receiver operating characteristic curve, and model fit was summarized using McFadden’s pseudo-R^2^. A *p*-value <0.05 was considered statistically significant.

## 3. Results

### 3.1. Microbial Composition and Taxonomic Distribution

A total of 248 bacterial isolates were detected from the 12 gym devices over the 5-day sampling period, encompassing 32 distinct families and 61 unique species (Table 2). Most isolates (*n* = 178, 71.8%) belonged to three families: Micrococcaceae (63 isolates, 25.4%), Staphylococcaceae (60 isolates, 24.2%), and Moraxellaceae (55 isolates, 22.2%). All three families are predominantly associated with human skin microbiota, although some constituent species (e.g., *Micrococcus luteus*) are also ubiquitous in environmental reservoirs. Within these dominant families, *Micrococcus luteus* (*n* = 60) was the single most frequently isolated species, followed by *Acinetobacter lwoffii* (*n* = 47) and *Staphylococcus saprophyticus* (*n* = 17). Clinically significant species were also detected, including *S. aureus* (*n* = 6), *Pseudomonas stutzeri* (*n* = 2), *Enterococcus faecalis* (*n* = 2), and *Stenotrophomonas maltophilia* (*n* = 4), albeit at substantially lower frequencies.

### 3.2. Temporal Dynamics of Contamination and Community Stability

The distribution of microbial burden and diversity varied considerably across time, weekday, and device categories (Table 3). Temporally, the highest number of positive detections occurred during the noon (91 isolates, 36.7%) and evening (89 isolates, 35.9%) sampling periods, while morning samples, collected after overnight cleaning, yielded the fewest isolates (68, 27.4%). Across the five-day sampling week, Monday and Friday exhibited the highest isolate counts (52 and 58 isolates, respectively; 21.0% and 23.4% of total), whereas Tuesday through Thursday showed lower yields (39–55 isolates, 15.7–22.2%). Device-level stratification revealed substantial heterogeneity. The highest numbers of positive detections were recorded from Device 1 (functional trainer; 26 isolates, 10.5%), Device 10 (seated row; 24 isolates, 9.7%), and Device 12 (treadmill; 22 isolates, 8.9%). Conversely, the lowest isolate counts were observed for Device 9 (arm ergometer; 16 isolates, 6.4%) and Device 4 (pulldown combo; 18 isolates, 7.2%).

**Figure 1:**
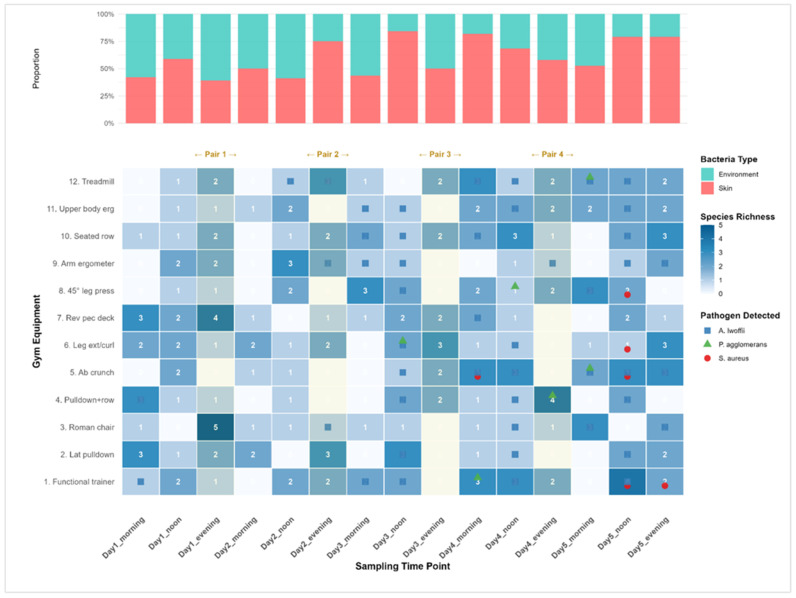
Temporal dynamics and taxonomic composition of microbial communities on gym equipment. Comprehensive temporal heatmap illustrating microbial shifts across 12 gym devices over a 5-day period (*n* = 180 samples). The upper bar plot shows the relative proportion of skin-associated (pink) versus environmental (teal) bacteria. The central heatmap displays species richness (color intensity and numerical values), with specific symbols denoting the detection of key pathogens: *Acinetobacter lwoffii* (blue square), *Pantoea agglomerans* (green triangle), and *Staphylococcus aureus* (red circle). Shaded regions indicate evening-to-morning cleaning intervals.

Analysis of the temporal distribution of microbial contaminants revealed a dynamic shift in community composition throughout the diurnal cycle (Figure 1). The proportion of skin-associated bacteria (pink) exhibited a marked increase as the day progressed, frequently peaking during evening sampling points. This trend was most pronounced on Day 5, where skin-associated taxa accounted for approximately 75% of the total microbial proportion across all devices. Species richness varied significantly across equipment types and time points, ranging from 0 to 5 distinct species per sample. High-contact equipment, specifically the Roman chair and functional trainer, demonstrated the highest peaks in species richness (reaching values of 5 and 4, respectively). While cleaning intervals (indicated by shaded pairs) generally resulted in a reduction in species richness by the following morning, microbial presence was rarely eliminated entirely, suggesting either incomplete disinfection or rapid re-colonization. The detection of clinically relevant pathogens was non-uniform. *Acinetobacter lwoffii* was the most ubiquitous pathogen, appearing consistently across various devices and time points. Notably, *S. aureus* detections were clustered primarily in the latter half of the study (Days 4 and 5), appearing on high-touch surfaces such as the functional trainer, ab crunch, and 45° leg press, coinciding with the highest observed proportions of skin-associated bacteria.

**Figure 2:**
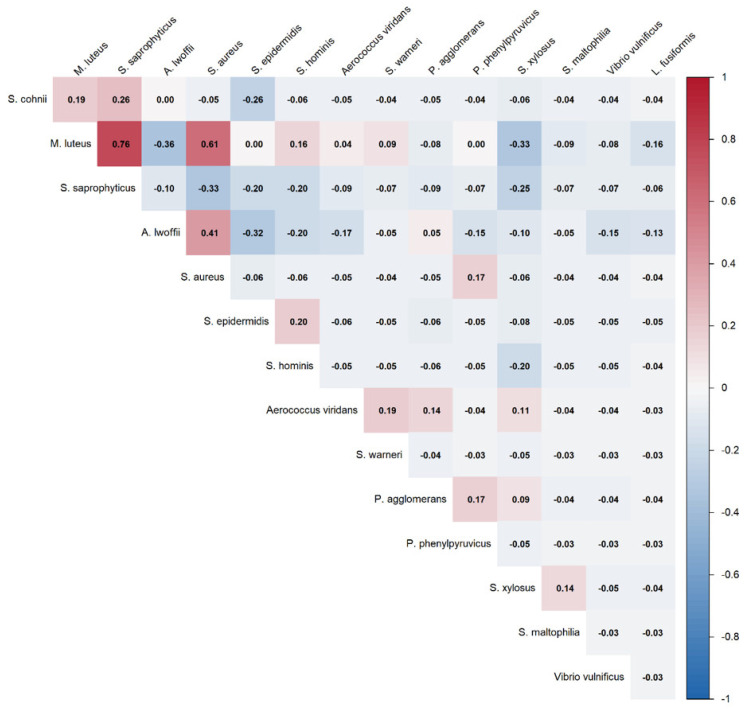
Microbial inter-species co-occurrence network. Heatmap illustrates the dominant species co-occurrence patterns across all gym equipment based on pairwise Phi correlation coefficient, indicating the strength and direction of association. Red cells indicate positive associations (co-occurrence; Phi > 0), while blue cells indicate negative associations (mutual exclusion; Phi < 0).

To determine whether specific microbial species form stable communities or guilds on gym surfaces, we performed a co-occurrence network analysis (Figure 2). The analysis revealed several high-strength positive associations. The strongest correlation was observed between *Micrococcus luteus* and *Staphylococcus saprophyticus* (Phi = 0.76), followed by a notable association between *Micrococcus luteus* and *S. aureus* (Phi = 0.61). Negative associations were less pronounced but still present; *Micrococcus luteus* and *Acinetobacter lwoffii* exhibited a negative correlation (Phi = −0.36), as did *Micrococcus luteus* and *Staphylococcus xylosus* (Phi = −0.33).

### 3.3. Device-Level Variation in Contamination and Risk Determinants

**Figure 3:**
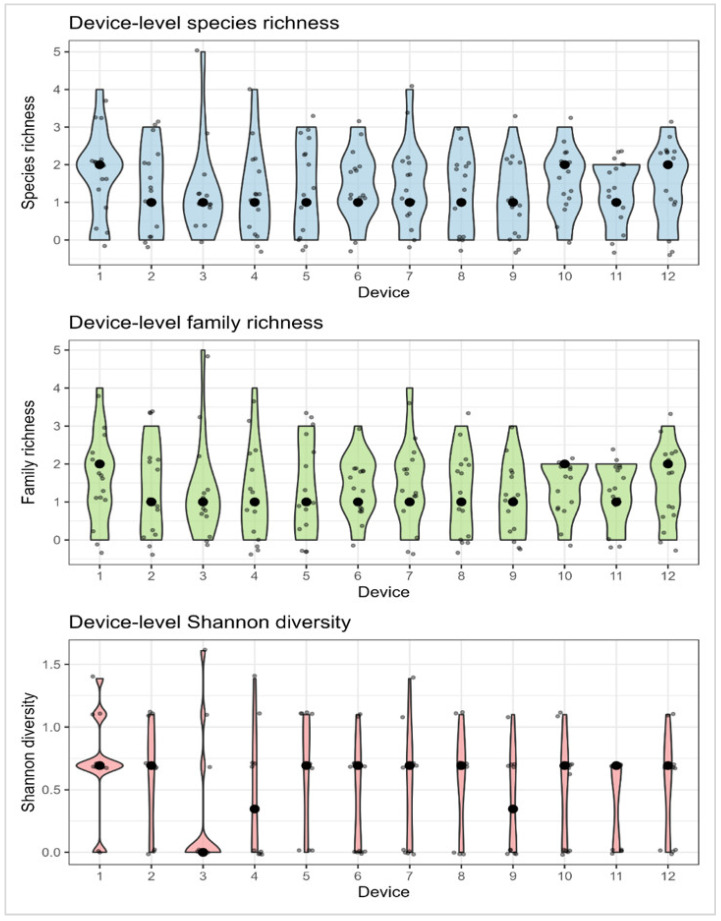
Comparative analysis of microbial richness and diversity across gym equipment. Violin plots of device-level microbial diversity, presented as species richness (**top**), family richness (**middle**), and Shannon diversity (**bottom**). Each plot shows the kernel density distribution, with individual sample points (gray) and median values (black circles) overlaid.

Microbial diversity was assessed across the 12 gym devices using three complementary metrics: species richness, family richness, and the Shannon diversity index (Figure 3). Species richness varied numerically across devices, ranging from a mean of 2.0 (Device 1: functional trainer) to 4.5 (Device 3: Roman chair and Device 4: pulldown combo). Family-level richness followed a nearly identical pattern, with the same devices showing the highest and lowest values. However, these apparent differences did not translate into statistically significant variation in overall community diversity as measured by the Shannon index. The Shannon diversity index, which incorporates both the number of taxa present and their relative evenness, was remarkably consistent across all 12 devices, with mean values clustering tightly around 1.0 (range: approximately 0.8 to 1.2 across devices). A Kruskal–Wallis non-parametric analysis of variance confirmed the absence of significant device-level heterogeneity in Shannon diversity (*p* = 0.44).

**Figure 4:**
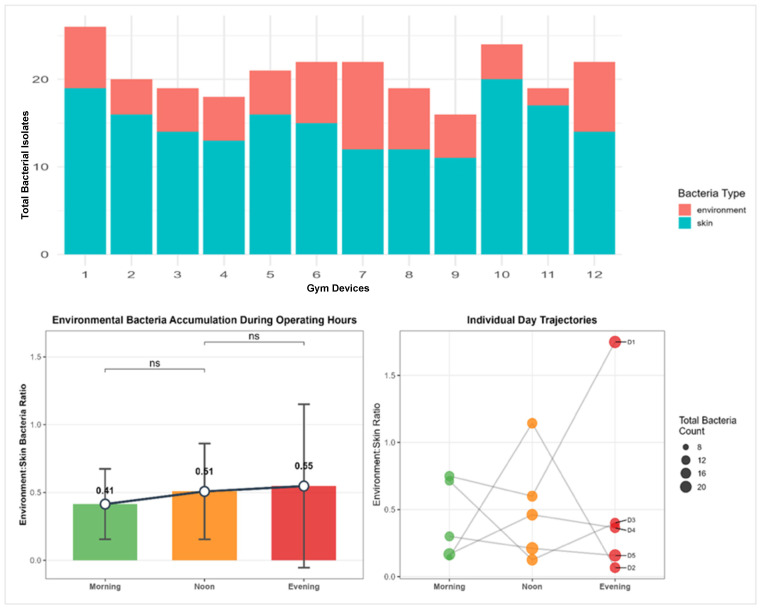
Distribution of skin-associated and environmental bacteria across gym devices and their temporal contamination dynamics. (**Top**): Stacked bar chart displaying the total count of bacterial isolates classified as skin flora versus environmental bacteria detected from each of the 12 gym devices. Data represent aggregate counts across all time points and sampling phases. (**Bottom left**): Environment–skin bacteria ratio across three time periods: morning, noon, and evening, with error bars representing 95% confidence intervals. (**Bottom right**): Individual day trajectories showing the change in environment–skin bacteria ratio from morning to evening for each of the five sampling days.

The relative contribution of skin-associated versus environmental bacteria showed minor variations across the 12 gym devices evaluated (Figure 4). Irrespectively, the microbial baseline of the equipment is generally defined by human contact rather than the ambient building environment. To investigate whether the microbial community resets or shifts its ecological origin throughout the day, the ratio of environmental to skin-associated bacteria was calculated. The aggregate data showed a slight but statistically non-significant upward trend in the ratio from morning (0.41) to evening (0.55); a Friedman test confirmed that these diurnal fluctuations were not statistically significant (*p* = 0.819). Despite this stability, individual day trajectories revealed high variability. Day 1 exhibited a sharp spike in the environment-to-skin ratio by the evening sampling point, coinciding with a high total bacteria count. In contrast, other days remained consistently low, with skin-associated bacteria maintaining a dominant presence (ratio < 0.5) throughout the day. Regardless of the time, the ratio rarely exceeded 1.0, confirming that skin-associated taxa remained the majority constituents of the gym microbiome from opening to closing. These classifications were based on the predominant ecological origin of each species in the context of human-associated indoor microbiota; however, some taxa (e.g., *Micrococcus luteus*) are ecologically versatile and may also occur widely in environmental reservoirs.

A device specific risk profile was constructed to stratify gym equipment according to contamination burden, pathogen prevalence, and temporal persistence (Table 4). The functional trainer and leg extension emerged as the highest risk devices, both classified as medium–high with composite risk scores of 42.3. These devices exhibited high overall positivity (80.0% and 86.7%, respectively), elevated *Staphylococcus* prevalence (33.3% and 53.3%), and frequent multi species detections (66.7% and 46.7%). The seated row machine and ab crunch machine followed closely, with risk scores of 39.7 and 35.7, respectively, placing them in the medium category. Notably, the ab crunch machine demonstrated a statistically significant increasing trend in contamination over the 5-day sampling period (*p* trend = 0.012, increasing), indicating a progressive increase in post-cleaning residual contamination levels across consecutive days. Conversely, the arm ergometer and pulldown combo were categorized as low risk, with composite scores of 23.3 and 25.0, respectively. The upper body ergometer, despite a moderate risk score (32.0, medium), exhibited a highly significant increasing temporal trend (*p*-trend = 0.006, increasing), suggesting that its contamination levels may escalate with continued use over consecutive days.

**Figure 5:**
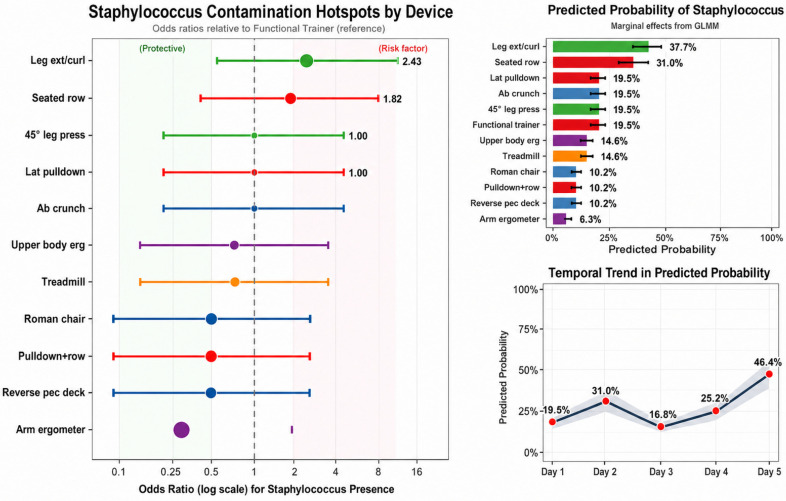
Device-specific Staphylococcus contamination hotspots and temporal trends. Analysis was based on 180 total samples, of which 54 (30.0%) were positive for *Staphylococcus*. (**Left**): Forest plot displaying adjusted odds ratios (ORs) for the presence of any *Staphylococcus* species across 12 gym devices, using the functional trainer as the reference category (OR = 1.00; dashed vertical line). Estimates were derived from a generalized linear mixed model (GLMM) with a binomial distribution, adjusting for time and weekday. Bars represent 95% confidence intervals. (**Top right**): Predicted probabilities of *Staphylococcus* detection for each device, calculated as marginal effects from the GLMM while holding other predictors constant. (**Bottom right**): Temporal trend in the predicted probability of *Staphylococcus* contamination over the 5-day sampling period, with observed daily positivity rates overlaid for comparison.

The distribution of *Staphylococcus* contamination varied markedly across gym equipment types (Figure 5). Using the functional trainer as the reference category (predicted probability = 19.5%), the GLMM identified several devices with substantially elevated odds of *Staphylococcus* detection. The leg extension/curl machine exhibited the highest risk, with an odds ratio of 2.43 (protective/risk factor notation suggests OR > 1 indicates elevated risk) and a predicted probability of 37.7%, nearly double that of the reference device. The seated row machine also showed elevated risk (OR = 1.82) with a predicted probability of 31.0%. The temporal analysis revealed a distinct mid-week peak in *Staphylococcus* contamination. The temporal analysis revealed a non-linear but overall upward trend in contamination risk. By Day 5, the predicted probability reached its peak at 46.4%, representing a nearly 2.4-fold increase from the study’s commencement (19.5% on Day 1).

Multivariable logistic regression was conducted to identify independent predictors of bacterial contamination on gym equipment surfaces (Table 5). Among the temporal factors evaluated, time of day emerged as a highly significant overall predictor (LRT *p* < 0.001). Relative to morning sampling (reference), the noon time point was associated with a markedly elevated odds of contamination (adjusted OR = 7.19, 95% CI: 2.59–23.73, *p* < 0.001). In contrast, the evening period did not differ significantly from morning (OR = 1.52, 95% CI: 0.68–3.43, *p* = 0.31). Neither weekday nor device type demonstrated significant overall effects in the multivariable model (LRT *p* = 0.218 and *p* = 0.809, respectively). The model demonstrated acceptable discrimination (AUC = 0.756) and explained a modest proportion of variance (McFadden R^2^ = 0.139).

## 4. Discussion

This study provides a high-resolution characterization of cultivable bacterial contamination across gym equipment, integrating taxonomic composition, temporal dynamics, ecological origin, and device-specific contamination profiling. Three principal findings emerged. First, the cultivable bacterial community recovered from gym surfaces was overwhelmingly dominated by skin-associated taxa, particularly members of Micrococcaceae, Staphylococcaceae, and Moraxellaceae, supporting direct human contact as the primary driver of contamination. Second, microbial burden and composition exhibited a pronounced temporal structure, with peak contamination occurring during the midday and evening periods and partial but incomplete reset following routine cleaning. Third, while overall microbial diversity remained relatively stable across devices, marked heterogeneity was observed in contamination persistence, multi-species burden, and *Staphylococcus* distribution, enabling the identification of device-specific contamination profiles and localized high-contact hotspots.

### 4.1. Microbial Composition and Taxonomic Patterns

The predominance of skin-associated taxa aligns with established paradigms in indoor microbial ecology, where human occupancy is the primary determinant of surface microbial composition [17]. The high frequency of *Micrococcus luteus*, *Acinetobacter lwoffii*, and coagulase-negative staphylococci reflects their ubiquity as skin commensals readily transferred via contact and capable of persisting on abiotic surfaces [29,30]. These findings align with previous taxonomical analyses reporting the dominance of the phyla Firmicutes, Proteobacteria, and Actinobacteria (as taxonomic groups that encompass the major genera identified in the present study) with diverse representation across bacterial families and genera [1]. Studies in fitness environments have documented substantial contamination of high-touch surfaces, including *S. aureus* detection on up to 38% of samples [4], although variability across facilities highlights the influence of hygiene practices and environmental conditions [4]. However, because the present study relied exclusively on culture-based methods, the detected microbial composition likely underrepresents the full diversity of gym-associated microbiota, particularly fastidious, slow-growing, or non-culturable organisms that may be identifiable using culture-independent molecular approaches. Accordingly, the reported taxonomic patterns should be interpreted as representing the cultivable fraction of the bacterial community present on gym surfaces rather than the complete microbial community. Furthermore, the present analysis was restricted to environmental surface samples and did not evaluate associations between human-associated microbiota and microorganisms detected on gym equipment surfaces; therefore, potential microbial transfer pathways could not be directly assessed.

While many detected organisms are benign commensals, the presence of clinically relevant species such as *S. aureus*, *Enterococcus faecalis*, and *Stenotrophomonas maltophilia* underscores the dual role of gym environments as reservoirs of both commensals and opportunistic pathogens [6,31]. In this study, the relatively low but consistent detection of *S. aureus*, along with its clustering on high-contact equipment later in the week, suggests contamination driven by cumulative user interactions and localized transmission dynamics. Although this finding is consistent with the established role of fomites in *S. aureus* transmission, particularly in community and athletic settings [1,13,32,33], environmental detection alone may not establish transmission risk or infection potential, which depends on additional factors such as infectious dose, transmission route, host susceptibility, and pathogen virulence [34,35]. The detection of environmental species, including *Pseudomonas* spp., *Stenotrophomonas maltophilia*, and *Vibrio vulnificus*, further indicates contributions from non-human sources. Although less abundant, these organisms may pose risks to immunocompromised individuals or reflect environmental factors such as inadequate ventilation or moisture control. However, given the relatively limited detection frequency and absence of direct transmission or infection data, these findings should be interpreted cautiously and viewed primarily as indicators of potential microbial exposure rather than a substantial public health risk.

### 4.2. Temporal Dynamics of Contamination and Community Stability

The pronounced diurnal variation in contamination, with significantly elevated odds of detection at noon, indicates rapid microbial accumulation driven by human activity. This aligns with studies showing that indoor microbial loads fluctuate dynamically with occupancy density and contact frequency [16,17,36]. The strong independent effect of time of day (*p* < 0.001), particularly the nearly sevenfold increase in contamination odds at noon versus morning, underscores the importance of temporal dynamics in surface microbiology and suggests that a single overnight cleaning, while necessary, is insufficient to maintain low contamination throughout operational hours. Importantly, while direct time-resolved occupancy data were not recorded, the predefined morning, noon, and evening sampling windows reflect systematic differences in gym usage intensity inherent to the facility’s operational schedule, thereby providing a structured proxy for occupancy-related variation across the day.

Persistence of contamination despite overnight cleaning indicates that routine disinfection reduces but does not eliminate microbial burden, consistent with evidence that many bacteria survive on dry surfaces and are rapidly reintroduced via human contact [5,6]. The sustained species richness in morning samples further suggests either the presence of persistent surface-associated communities or a limited residual efficacy of disinfectants, allowing rapid recolonization from the environment. This suggests a dynamic equilibrium in which microbial communities are continuously disrupted and re-established [37].

The observed increase in *Staphylococcus* contamination over time supports cumulative contamination effects, but should be interpreted with caution. Although detections were more frequent on Days 4–5, user-level information and usage patterns were not collected, and the number of *S. aureus* detections was limited; therefore, this pattern may simply reflect normal day-to-day variation rather than a systematic temporal trend. Similar multi-day build-up patterns (i.e., increasing baseline contamination across consecutive cleaning cycles) have been reported in other high-contact environments, including hospitals and public transport systems [38,39]. However, because the gym was already in routine operation before sampling began, Day 1 does not represent a true baseline. As a result, contamination detected on later days cannot be assumed to reflect accumulation during the study period, and the observed pattern should be viewed as a descriptive fluctuation rather than as evidence of progressive accumulation.

These temporal dynamics are consistent with diurnal variation in gym usage patterns that likely reflect fluctuations in human occupancy intensity and the “humanization” of the microbial environment. Even after morning cleaning, the low environment-to-skin ratio (0.41) indicates incomplete restoration of a non-human microbial state. This pattern is consistent with prior work showing that human occupancy rapidly reshapes indoor microbial communities toward skin-associated taxa on frequently touched surfaces [17]. Despite diurnal fluctuations in contamination probability, the stable environment-to-skin ratio (*p* = 0.8) suggests a rapid attainment of a steady-state community dominated by skin microbiota. While overnight cleaning effectively reduces environmental bacteria, the persistent presence of skin-associated taxa (≥45%) highlights their resilience. Accordingly, although midday cleaning may moderate peak contamination, enhanced overnight disinfection combined with targeted attention to high-contact equipment is likely to produce the greatest reductions in surface microbial burden.

The co-occurrence network analysis shows that contamination reflects multi-species assemblages rather than isolated taxa, consistent with the notion that human skin microbiota are transferred to built environments as structured, cohesive communities [40]. Positive associations between dominant taxa largely reflect shared origin and co-transfer via human contact, whereas weaker negative associations may indicate niche differentiation or environmental tolerance differences. However, given the cross-sectional, culture-based design, these associations should be interpreted cautiously, as co-detection does not imply direct biological interaction. Beyond shared source attribution, the co-occurrence network provides a structured view of non-random co-detection patterns, highlighting taxa that repeatedly co-occur under real-world exposure conditions and may therefore reflect stable exposure-related assemblages rather than independent occurrence. Positive correlations between *Micrococcus luteus* and multiple *Staphylococcus* species, including *S. aureus*, suggest frequent co-deposition as part of a shared “skin microbiome package”, with *Micrococcus luteus* potentially serving as a sentinel indicator of staphylococcal contamination. These associations should be understood strictly as reflecting repeated co-presence on sampled surfaces, not coordinated ecological behavior or interaction. The tight clustering of *Staphylococcus epidermidis*, *Staphylococcus hominis*, and *Micrococcus luteus* further supports joint transfer via skin shedding and contact, consistent with prior evidence that *Staphylococcus* spp. and *Micrococcus luteus* represent dominant co-shed skin taxa [29,41]. In addition, the exclusive use of culture-based detection limits the interpretation of ecological interactions and functional characteristics, as non-culturable taxa and strain-level traits were not assessed. Consequently, the inferred community relationships should be interpreted as partial co-detection patterns rather than comprehensive microbiome interactions. Overall, the network analysis identifies statistically non-random co-occurrence patterns but does not provide evidence of mechanistic, ecological, or biological interactions among taxa.

Negative associations between environmental taxa and skin commensals likely reflect ecological displacement as surfaces become increasingly “humanized”, potentially involving competition or habitat differentiation, particularly for *S. aureus* [42]. Such dynamics between resident skin microbiota and environmental organisms have been previously described in exposed surface ecosystems [43]. Environmental species such as *Vibrio vulnificus* and *Pantoea agglomerans* occupy peripheral network positions, consistent with transient introduction rather than stable colonization. Overall, the network structure indicates the dominance of co-transferred skin-associated assemblages, suggesting that interventions targeting representative commensals (e.g., *Staphylococcus epidermidis*) may reduce broader Gram-positive burden, while *S. aureus* requires specific monitoring due to its independent and clinically relevant behavior.

### 4.3. Device-Level Variation in Contamination Patterns and Determinants

Although Shannon diversity remained stable across gym devices, indicating broadly similar ecological complexity, differences in contamination structure were evident. The absence of significant variation in Shannon diversity despite differences in species richness suggests relatively even and compositionally similar microbial communities across equipment. This apparent homogenization aligns with studies showing that human-mediated dispersal and frequent contact in built environments drive microbial mixing and convergence across surfaces [17,44]. Higher richness (e.g., Roman chair, pulldown combo) likely reflects transient or rare taxa rather than distinct communities. Overall, this supports extensive cross-contamination through user movement, producing a largely uniform microbial assemblage. Environmental factors such as air circulation and cleaning protocols likely exert greater influence on microbial composition than device-specific characteristics [36,45], resulting in relatively diffuse exposure patterns across equipment. The dominance of skin-associated bacteria across devices further indicates that surface microbiota primarily reflects human shedding during exercise.

Despite this ecological uniformity, contamination patterns were unevenly distributed across equipment types. High-risk devices showed higher positivity rates, greater *Staphylococcus* prevalence, increased multi-species burden, and evidence of persistence, indicating that risk is driven more by dominant taxa enrichment than overall diversity, consistent with the ecological principles of functional dominance [37]. The functional trainer, leg extension/curl, and seated row likely act as high-contact hubs due to prolonged use, intensive skin contact, and material properties (e.g., vinyl surfaces retaining moisture and debris), which can enhance microbial persistence, particularly of *Staphylococcus* spp. Prior studies similarly show that surface material, contact frequency, and user behavior strongly influence microbial transfer and persistence [46,47]. In the present study, however, device-specific contamination profiles should be interpreted as relative indicators of contamination burden rather than as formal assessments of infection risk, which would require quantitative microbial load data, infectious dose thresholds, and exposure modeling. In addition, the composite contamination index used to summarize device-level patterns should be interpreted with caution, as it represents an exploratory, conceptually derived framework and has not undergone external validation.

The lack of a statistically significant device effect in multivariable models may reflect limited statistical power or residual heterogeneity; however, consistent patterns across analyses support meaningful device-level variation. The modest pseudo-R^2^ further indicates that additional factors, such as surface porosity, user behavior, and ambient humidity, likely contribute to contamination dynamics and warrant further investigation. This aligns with evidence that environmental conditions and human interaction patterns often outweigh material properties in determining indoor microbial loads [18,31]. Accordingly, the identified device-specific contamination patterns should be interpreted as relative comparative trends within the studied facility rather than universally generalizable risk rankings across fitness environments or formal assessments of infection risk.

Temporal trends further refined risk patterns, with *Staphylococcus* contamination increasing across the week and peaking on Friday (46.4%), indicating incomplete microbial reset by daily cleaning. This pattern was most pronounced on the ab crunch and upper body ergometer, while lower persistence on the lat pulldown suggests variable cleaning efficacy. A similar trend has been reported in high-touch environments where inconsistent disinfection allows for progressive microbial build-up, particularly of resilient taxa such as *Staphylococcus* spp. [48]. The contamination profiling framework identified the leg extension/curl and seated row as relative contamination hotspots that may benefit from intensified cleaning. Overall, while midday cleaning may reduce peak contamination, optimizing cleaning frequency and targeting high-risk equipment is likely more effective than device-specific interventions alone.

### 4.4. Limitations and Strengths

Some limitations warrant consideration. The cross-sectional design precludes causal inference, and reverse causation cannot be excluded. Culture-based methods likely underestimate total microbial diversity, particularly for non-culturable organisms [49]. The absence of DNA-based methods (e.g., 16S rRNA sequencing, metagenomics) limits the ability to characterize the full microbial community and associated functional traits, including antimicrobial resistance determinants. Consequently, the findings should be interpreted as representing the cultivable fraction of the bacterial community rather than the complete gym microbiome. However, it should also be recognized that standard DNA-based approaches generally do not distinguish between viable and non-viable microorganisms; therefore, culture-based and molecular methods provide complementary perspectives on environmental microbial contamination. Moreover, quantitative microbial load measurements (e.g., total viable counts or pathogen concentrations) were not performed, and fungal microorganisms were not included in the analysis, thereby narrowing the scope of contamination characterization. Sampling was conducted within a single fitness facility, which may limit the external validity and generalizability of the findings, because microbial contamination profiles may be influenced by facility-specific factors, including user demographics, occupancy patterns, cleaning protocols, ventilation characteristics, and equipment composition. In addition, the detection of opportunistic pathogens does not equate to infection risk, as transmission depends on host susceptibility, inoculum size, and pathogen virulence [31]. Finally, behavioral variables such as hand hygiene practices, equipment cleaning by users, and duration of contact were not directly measured, precluding adjustment for key exposure determinants.

Despite these limitations, the study has several notable strengths. The repeated-measures design with structured intra-day and inter-day sampling provides high temporal resolution, enabling the robust characterization of dynamic contamination patterns rarely captured in prior studies. The inclusion of a diverse set of gym equipment enhances ecological validity and allows for nuanced device-level comparisons. The integration of taxonomic identification using MALDI-TOF MS ensures high diagnostic accuracy at the species level, strengthening the reliability of microbiological findings. Importantly, the study extends beyond prevalence estimates by integrating ecological metrics, source attribution, co-occurrence analysis, and multivariable modeling, alongside a composite device-specific risk framework that translates microbiological data into actionable risk stratification.

### 4.5. Implications for Public Health and Future Research

The findings highlight human contact as the primary driver of gym surface contamination, supporting the importance of consistent hygiene practices, particularly hand hygiene and routine disinfection of frequently touched equipment. The observed microbial profile supports a targeted hygiene approach combining the routine disinfection of high-contact surfaces with periodic deep cleaning to address environmental reservoirs and potentially persistent taxa. Identification of high-risk devices further enables the prioritization of cleaning efforts, improving efficiency, especially in resource-constrained settings.

Future research should adopt longitudinal and interventional designs to evaluate the effectiveness of cleaning strategies and user compliance. Incorporation of molecular methods, such as 16S rRNA sequencing and metagenomics, together with quantitative microbial load measurements, would allow for a more comprehensive characterization of microbial communities, resistance patterns, and contamination dynamics [27,50]. Additionally, studies linking environmental contamination to colonization or infection outcomes are needed to better define clinical relevance.

## 5. Conclusions

In this repeated-measures cross-sectional study, microbial contamination on gym equipment was predominantly driven by human-derived skin microbiota, exhibiting pronounced temporal dynamics and clear device-specific risk heterogeneity. Despite relatively stable overall microbial diversity across devices, contamination accumulated progressively during daily use and was characterized by localized enrichment of opportunistic pathogens and identifiable *Staphylococcus* hotspots on high-contact equipment. However, the study relied on culture-based methods; therefore, the findings reflect only the cultivable fraction of the microbial community and should not be interpreted as representing the complete gym microbiome or serving as a basis for infection risk assessment.

The relatively limited detection of clinically significant pathogens suggests that the observed contamination primarily reflects routine microbial transfer associated with human occupancy rather than evidence of substantial infection risk. These findings indicate that contamination risk is governed more by temporal exposure patterns and contact intensity than by ecological diversity alone. Collectively, the findings identify gym environments as dynamic reservoirs of human-associated microbes and potential exposure pathways, underscoring the need for optimized, targeted hygiene strategies. These include increased cleaning frequency during peak usage and the prioritization of high-contact devices, and they provide a basis for future research on microbial transmission in public indoor settings.

## Figures and Tables

**Table 1:** Gym equipment sampled for handle surface microbiological assessment.

Device ID	Device Type	Primary Contact Area
1	Functional trainer (wire rope hoist)	Adjustable pulley handles
2	Lat pulldown (wire rope hoist)	Pulldown bar grips
3	Roman chair	Front stabilization handles
4	Pulldown + seated row combo	Pulley grips/row handle
5	Ab crunch machine	Side/overhead support grips
6	Leg extension	Side stabilization handles
7	Reverse pec deck (butterfly reverse)	Horizontal handles
8	45° leg press	Side safety handles
9	Arm ergometer (wheel 1)	Rotary handles
10	Seated row machine (wheel 2)	Row handle
11	Upper body ergometer	Crank handles
12	Treadmill	Front handrails

**Table 2:** Distribution of bacterial families, species, and ecological origins.

Bacterial Family	Ecological Origin	Species	Species Isolates	Family Total
Micrococcaceae	Skin	*Micrococcus luteus*	60	63 (25.4%)
		*Arthrobacter cumminsii*	1	
		*Kocuria rosea*	1	
		*Kocuria varians*	1	
Staphylococcaceae	Skin	*Staphylococcus saprophyticus*	17	60 (24.2%)
		*Staphylococcus epidermidis*	9	
		*Staphylococcus xylosus*	8	
		*Staphylococcus cohnii*	7	
		*Staphylococcus hominis*	7	
		*Staphylococcus aureus*	6	
		*Staphylococcus warneri*	4	
		*Staphylococcus arlettae*	2	
Moraxellaceae	Skin	*Acinetobacter lwoffii*	47	55 (22.2%)
		*Psychrobacter phenylpyruvicus*	4	
		*Acinetobacter johnsonii*	2	
		*Acinetobacter ursingii*	2	
Pseudomonadaceae	Environment	*Pseudomonas stutzeri*	2	8 (3.2%)
		*Pseudomonas oryzihabitans*	3	
		*Pseudomonas luteola*	1	
		*Pseudomonas putida*	1	
		*Pseudomonas fluorescens*	1	
Enterobacteriaceae	Environment	*Pantoea agglomerans*	6	7 (2.8%)
		*Serratia odorifera*	1	
Bacillaceae	Environment	*Lysinibacillus fusiformis*	2	6 (2.4%)
		*Bacillus simplex*	2	
		*Bacillus*	1	
		*Lysinibacillus*	1	
Aerococcaceae	Environment	*Acerococcus viridans*	5	5 (2.0%)
Enterococcaceae	Environment	*Enterococcus faecalis*	2	4 (1.6%)
		*Enterococcus faecium*	2	
Streptococcaceae	Environment	*Streptococcus constellatus*	2	4 (1.6%)
		*Lactococcus lactis*	1	
		*Streptococcus pneuminiae*	1	
Vibrionaceae	Environment	*Vibrio vulnificus*	4	4 (1.6%)
Xanthomonadaceae	Environment	*Stenotrophomonas maltophilia*	4	4 (1.6%)
Brevibacteriaceae	Environment	*Brevibacterium casei*	1	2 (0.8%)
		*Cellulosimicrobium cellulans*	1	
Brucellaceae	Environment	*Ochrobactrum anthropi*	2	2 (0.8%)
Carnobacteriaceae	Environment	*Granulicatella adiacens*	1	2 (0.8%)
		*Carnobacterium divergens*	1	
Caulobacteraceae	Environment	*Brevundimonas diminuta*	1	2 (0.8%)
		*Brevundimonas vesicularis*	1	
Cellulomonadaceae	Environment	*Oerskovia turbata*	2	2 (0.8%)
Dermacoccaceae	Environment	*Kytococcus sedentarius*	1	2 (0.8%)
		*Dermacoccus nishinomiyaensis*	1	
Lactobacillaceae	Environment	*Alloiococcus otitis*	1	2 (0.8%)
		*Lactobacillus delbrueckii*	1	
Microbacteriaceae	Environment	*Microbacterium arborescens*	1	2 (0.8%)
		*Leifsonia aquatica*	1	
Alcaligenaceae	Environment	*Alcaligenes faecalis*	1	1 (0.4%)
Clostridiaceae	Environment	*Clostridium sporogenes*	1	1 (0.4%)
Coriobacteriaceae	Environment	*Collinsella aerofaciens*	1	1 (0.4%)
Leuconostocaceae	Environment	*Leuconostoc mesenteroides*	1	1 (0.4%)
Listeriaceae	Environment	*Listeria grayi*	1	1 (0.4%)
Methylobacteriaceae	Environment	*Methylobacterium mesophilicum*	1	1 (0.4%)
Neisseriaceae	Environment	*Eikenella corrodens*	1	1 (0.4%)
Nocardiaceae	Environment	*Rhodococcus erythropolis*	1	1 (0.4%)
Paenibacillaceae	Environment	*Paenibacillus pabuli*	1	1 (0.4%)
Pasteurellaceae	Environment	*Haemophilus haemolyticus*	1	1 (0.4%)
Propionibacteriaceae	Skin	*Cutibacterium acnes*	1	1 (0.4%)
Rhizobiaceae	Environment	*Rhizobium radiobacter*	1	1 (0.4%)

**Table 3:** Microbial characteristics by time, weekday, and device.

Stratification	Subgroup	Samples	Positive Detections	Species	Families
Overall	-	180	248	61	32
Time	Morning	60	68 (27.4%)	28 (45.9%)	14 (43.7%)
	Noon	60	91 (36.7%)	31 (50.8%)	22 (68.7%)
	Evening	60	89 (35.9%)	33 (54.1%)	19 (59.4%)
Weekday	Monday	36	52 (21.0%)	32 (52.4%)	20 (62.5%)
	Tuesday	36	39 (15.7%)	16 (26.2%)	11 (34.4%)
	Wednesday	36	44 (17.4%)	16 (26.2%)	12 (37.5%)
	Thursday	36	55 (22.2%)	22 (36.1%)	14 (43.7%)
	Friday	36	58 (23.4%)	19 (31.1%)	11 (34.4%)
Device	1	15	26 (10.5%)	16 (26.2%)	9 (28.1%)
	2	15	20 (8.1%)	11 (18.0%)	7 (21.9%)
	3	15	19 (7.7%)	10 (16.4%)	7 (21.9%)
	4	15	18 (7.2%)	11 (18.0%)	8 (25.0%)
	5	15	21 (8.5%)	10 (16.4%)	8 (25.0%)
	6	15	22 (8.9%)	15 (24.6%)	10 (31.2%)
	7	15	22 (8.9%)	15 (24.6%)	11 (34.4%)
	8	15	19 (7.7%)	14 (22.9%)	10 (31.2%)
	9	15	16 (6.4%)	10 (16.4%)	9 (28.1%)
	10	15	24 (9.7%)	11 (18.0%)	6 (18.9%)
	11	15	19 (7.7%)	9 (14.7%)	5 (15.6%)
	12	15	22 (8.9%)	13 (21.3%)	10 (31.2%)

**Table 4:** Device-specific microbial risk profile on gym equipment.

Gym Equipment	Any Bacteria	Richness ^1^	*Staphylococcus*	Multi- Species	Persistence ^2^	*p*-Trend ^3^ & Direction	% Risk ^4^	Category
Functional trainer	80.0% (12/15)	1.7 ± 1.2	33.3% (5/15)	66.7% (10/15)	36.4%	0.221 (→ No trend)	42.3	Medium–High
Leg extension	86.7% (13/15)	1.5 ± 0.9	53.3% (8/15)	46.7% (7/15)	33.3%	0.569 (→ No trend)	42.3	Medium–High
Seated row machine	86.7% (13/15)	1.5 ± 0.9	46.7% (7/15)	53.3% (8/15)	33.3%	0.337 (→ No trend)	39.7	Medium
Ab crunch machine	66.7% (10/15)	1.4 ± 1.2	33.3% (5/15)	46.7% (7/15)	55.6%	0.012 (↑ Increasing)	35.7	Medium
Treadmill	80.0% (12/15)	1.5 ± 1.0	26.7% (4/15)	53.3% (8/15)	36.4%	0.146 (→ No trend)	33.3	Medium
45° leg press	66.7% (10/15)	1.3 ± 1.1	33.3% (5/15)	46.7% (7/15)	33.3%	0.140 (→ No trend)	33.3	Medium
Upper body ergometer	80.0% (12/15)	1.3 ± 0.8	26.7% (4/15)	46.7% (7/15)	36.4%	0.006 (↑ Increasing)	32.0	Medium
Lat pulldown	66.7% (10/15)	1.3 ± 1.2	33.3% (5/15)	46.7% (7/15)	22.2%	0.293 (→ No trend)	31.0	Medium
Reverse pec deck	80.0% (12/15)	1.5 ± 1.1	20.0% (3/15)	46.7% (7/15)	36.4%	0.072 (→ No trend)	30.3	Medium
Roman chair	80.0% (12/15)	1.3 ± 1.3	20.0% (3/15)	20.0% (3/15)	36.4%	0.787 (→ No trend)	25.0	Medium
Pulldown combo	66.7% (10/15)	1.2 ± 1.2	20.0% (3/15)	33.3% (5/15)	44.4%	0.886 (→ No trend)	25.0	Low
Arm ergometer	66.7% (10/15)	1.1 ± 1.0	13.3% (2/15)	33.3% (5/15)	44.4%	0.361 (→ No trend)	23.3	Low

^1^ Mean ± SD species per sample; ^2^ Percentage of consecutive samplings where ≥1 species persisted; ^3^ The linear trend test over five days: An upward arrow indicates a statistically significant increasing trend (*p*-trend < 0.05), whereas a rightward arrow indicates no statistically significant trend (*p*-trend ≥ 0.05); ^4^ Composite: 35% *S. aureus* + 25% *Staphylococcus* + 20% positivity + 20% multi-species.

**Table 5:** Multivariable logistic regression analysis of temporal and equipment-related predictors for surface bacterial contamination of gym equipment.

Predictor Category	Level/Metric	Odds Ratio	95% CI	Individual *p*-Value	Overall *p*-Value (LRT)
Time of Day	Morning	Reference	--	--	<0.001
	Noon	7.19	2.59–23.73	<0.001	
	Evening	1.52	0.68–3.43	0.31	
Weekday	Monday	Reference	--	--	0.218
	Tuesday	0.43	0.13–1.35	0.157	
	Wednesday	0.50	0.15–1.59	0.249	
	Thursday	1.56	0.42–6.11	0.509	
	Friday	0.70	0.21–2.27	0.548	
Device Type	Functional trainer	Reference	--	--	0.809
	Leg extension	1.70	0.22–15.69	0.611	
	Seated row	1.70	0.22–15.69	0.611	
	Roman chair	1.00	0.14–6.97	1.000	
	Reverse pec deck	1.00	0.14–6.97	1.000	
	Upper body erg	1.00	0.14–6.97	1.000	
	Treadmill	1.00	0.14–6.97	1.000	
	Lat pulldown	0.45	0.07–2.59	0.382	
	Pulldown combo	0.45	0.07–2.59	0.382	
	Ab crunch	0.45	0.07–2.59	0.382	
	Leg press	0.45	0.07–2.59	0.382	
	Arm ergometer	0.45	0.07–2.59	0.382	

CI: confidence interval; LRT: likelihood ratio test. Note: Odds ratio >1 indicates increased odds of contamination. Model performance demonstrated acceptable discrimination (AUC = 0.756) and fit (McFadden R^2^ = 0.139, AIC = 208.400).

## Data Availability

The data supporting the findings of this study are available from the corresponding author upon reasonable request.

## Abbreviations

The following abbreviations are used in this manuscript:

*S. aureus*	*Staphylococcus aureus*
MALDI-TOF MS	Matrix-assisted laser desorption and ionization time-of-flight mass spectrometry
CI	Confidence interval
OR	Odds ratio
AIC	Akaike information criterion
AUC	Area under the curve
GLMM	Generalized linear mixed model
LRT	Likelihood ratio test
µL	Microliter

## References

[1] Mukherjee N.A., Dowd S.E., Wise A., Kedia S., Vohra V., Banerjee P. (2014). Diversity of bacterial communities of fitness center surfaces in a U.S. metropolitan area. Int. J. Environ. Res. Public Health.

[2] Ryan K.A., Ifantides C., Bucciarelli C., Saliba H., Tuli S., Black E., Thompson L.A. (2011). Are gymnasium equipment surfaces a source of staphylococcal infections in the community?. Am. J. Infect. Control.

[3] Wood M., Gibbons S.M., Lax S., Eshoo-Anton T.W., Owens S.M., Kennedy S., Gilbert J.A., Hampton-Marcell J.T. (2015). Athletic equipment microbiota are shaped by interactions with human skin. Microbiome.

[4] Dalman M., Bhatta S., Nagajothi N., Thapaliya D., Olson H., Naimi H.M., Smith T.C. (2019). Characterizing the molecular epidemiology of *Staphylococcus aureus* across and within fitness facility types. BMC Infect. Dis..

[5] Kramer A., Assadian O. (2014). Survival of microorganisms on inanimate surfaces. Use of Biocidal Surfaces for Reduction of Healthcare Acquired Infections.

[6] Kramer A., Schwebke I., Kampf G. (2006). How long do nosocomial pathogens persist on inanimate surfaces? A systematic review. BMC Infect. Dis..

[7] Skowron K., Bauza-Kaszewska J., Kraszewska Z., Wiktorczyk-Kapischke N., Grudlewska-Buda K., Kwiecińska-Piróg J., Wałecka-Zacharska E., Radtke L., Gospodarek-Komkowska E. (2021). Human skin microbiome: Impact of intrinsic and extrinsic factors on skin microbiota. Microorganisms.

[8] Hou J., Nakajima M., Nishiuchi Y., Ogura D., Teramoto A., Kuratomi C., Iwamoto Y., Okamura Y., Moriguchi K., Dovjak M. (2025). Occupants and surface types drive microbial dynamics in controlled indoor environments. Environ. Microbiome.

[9] Cao L., Yang L., Swanson C.S., Li S., He Q. (2021). Comparative analysis of impact of human occupancy on indoor microbiomes. Front. Environ. Sci. Eng..

[10] Wißmann J.E., Kirchhoff L., Brüggemann Y., Todt D., Steinmann J., Steinmann E. (2021). Persistence of pathogens on inanimate surfaces: A narrative review. Microorganisms.

[11] Linz M.S., Mattappallil A., Finkel D., Parker D. (2023). Clinical impact of *Staphylococcus aureus* skin and soft tissue infections. Antibiotics.

[12] Knox J., Uhlemann A.-C., Lowy F.D. (2015). *Staphylococcus aureus* infections: Transmission within households and the community. Trends Microbiol..

[13] Zhang M., Ma Y., Xu H., Wang M., Li L. (2023). Surfaces of gymnastic equipment as reservoirs of microbial pathogens with potential for transmission of bacterial infection and antimicrobial resistance. Front. Microbiol..

[14] Viegas C., Peixoto C., Gomes B., Dias M., Cervantes R., Pena P., Slezakova K., Pereira M.D.C., Morais S., Carolino E. (2024). Assessment of Portuguese fitness centers: Bridging the knowledge gap on harmful microbial contamination with focus on fungi. Environ. Pollut..

[15] Liang Z., Dong C.B., Liang H., Zhen Y.X., Zhou R.L., Han Y.F., Liang Z.Q. (2022). A microbiome study reveals the potential relationship between the bacterial diversity of a gymnastics hall and human health. Sci. Rep..

[16] Chawla H., Anand P., Garg K., Bhagat N., Varmani S.G., Bansal T., McBain A.J., Marwah R.G. (2023). A comprehensive review of microbial contamination in the indoor environment: Sources, sampling, health risks, and mitigation strategies. Front. Public Health.

[17] Lax S., Smith D.P., Hampton-Marcell J., Owens S.M., Handley K.M., Scott N.M., Gibbons S.M., Larsen P., Shogan B.D., Weiss S. (2014). Longitudinal analysis of microbial interaction between humans and the indoor environment. Science.

[18] Kembel S.W., Jones E., Kline J., Northcutt D., Stenson J., Womack A.M., Bohannan B.J.M., Brown G.Z., Green J.L. (2012). Architectural design influences the diversity and structure of the built environment microbiome. ISME J..

[19] Cassol I., Ibañez M., Bustamante J.P. (2025). Key features and guidelines for the application of microbial alpha diversity metrics. Sci. Rep..

[20] Knights D., Kuczynski J., Charlson E.S., Zaneveld J., Mozer M.C., Collman R.G., Bushman F.D., Knight R., Kelley S.T. (2011). Bayesian community-wide culture-independent microbial source tracking. Nat. Methods.

[21] Callewaert C., Ravard Helffer K., Lebaron P. (2020). Skin microbiome and its interplay with the environment. Am. J. Clin. Dermatol..

[22] Faust K., Raes J. (2012). Microbial interactions: From networks to models. Nat. Rev. Microbiol..

[23] Burmølle M., Ren D., Bjarnsholt T., Sørensen S.J. (2014). Interactions in multispecies biofilms: Do they actually matter?. Trends Microbiol..

[24] Moissl-Eichinger C., Auerbach A.K., Probst A.J., Mahnert A., Tom L., Piceno Y., Andersen G.L., Venkateswaran K., Rettberg P., Barczyk S. (2015). Quo vadis? Microbial profiling revealed strong effects of cleanroom maintenance and routes of contamination in indoor environments. Sci. Rep..

[25] Patra J.K., Das G., Das S.K., Thatoi H. (2020). Isolation, culture, and biochemical characterization of microbes. A Practical Guide to Environmental Biotechnology.

[26] Timm C.M., Loomis K., Stone W., Mehoke T., Brensinger B., Pellicore M., Staniczenko P.P.A., Charles C., Nayak S., Karig D.K. (2020). Isolation and Characterization of Diverse Microbial Representatives from the Human Skin Microbiome. Microbiome.

[27] Gilbert J.A., Stephens B. (2018). Microbiology of the built environment. Nat. Rev. Microbiol..

[28] Li Z., Ju Y., Xia J., Zhang Z., Zhen H., Tong X., Sun Y., Lu H., Zong Y., Chen P. (2023). Integrated Human Skin Bacteria Genome Catalog Reveals Extensive Unexplored Habitat-Specific Microbiome Diversity and Function. Adv. Sci..

[29] Grice E.A., Segre J.A. (2011). The skin microbiome. Nat. Rev. Microbiol..

[30] Otto M. (2009). *Staphylococcus epidermidis*—The “accidental” pathogen. Nat. Rev. Microbiol..

[31] Otter J.A., Yezli S., Salkeld J.A., French G.L. (2013). Evidence that contaminated surfaces contribute to the transmission of hospital pathogens and an overview of strategies to address contaminated surfaces in hospital settings. Am. J. Infect. Control.

[32] Begier E.M., Frenette K., Barrett N.L., Mshar P., Petit S., Boxrud D.J., Watkins-Colwell K., Wheeler S., Cebelinski E.A., Glennen A. (2004). A high-morbidity outbreak of methicillin-resistant *Staphylococcus aureus* among players on a college football team, facilitated by cosmetic body shaving and turf burns. Clin. Infect. Dis..

[33] Naimi T.S., LeDell K.H., Como-Sabetti K., Borchardt S.M., Boxrud D.J., Etienne J., Johnson S.K., Vandenesch F., Fridkin S., O’Boyle C. (2003). Comparison of community- and health care-associated methicillin-resistant *Staphylococcus aureus* infection. JAMA.

[34] Stephens B., Azimi P., Thoemmes M.S., Heidarinejad M., Allen J.G., Gilbert J.A. (2019). Microbial Exchange via Fomites and Implications for Human Health. Curr. Pollut. Rep..

[35] Kramer A., Lexow F., Bludau A., Köster A.M., Misailovski M., Seifert U., Eggers M., Rutala W., Dancer S.J., Scheithauer S. (2024). How Long Do Bacteria, Fungi, Protozoa, and Viruses Retain Their Replication Capacity on Inanimate Surfaces? A Systematic Review Examining Environmental Resilience versus Healthcare-Associated Infection Risk by “Fomite-Borne Risk Assessment”. Clin. Microbiol. Rev..

[36] Hospodsky D., Qian J., Nazaroff W.W., Yamamoto N., Bibby K., Rismani-Yazdi H., Peccia J. (2012). Human occupancy as a source of indoor airborne bacteria. PLoS ONE.

[37] Shade A., Peter H., Allison S.D., Baho D.L., Berga M., Bürgmann H., Huber D.H., Langenheder S., Lennon J.T., Martiny J.B.H. (2012). Fundamentals of microbial community resistance and resilience. Front. Microbiol..

[38] Prasek K., Kiersnowska I., Wójkowska-Mach J., Różańska A., Romaniszyn D., Foryciarz E., Kwiećkowska L.B., Krzych-Fałta E. (2025). Microbial contamination on high-touch surfaces in outpatient clinics: Identification of bacterial strains from areas of patient and medical staff occupancy. Microorganisms.

[39] Ly Y.T., Leuko S., Moeller R. (2024). An overview of the bacterial microbiome of public transportation systems—Risks, detection, and countermeasures. Front. Public Health.

[40] Costello E.K., Lauber C.L., Hamady M., Fierer N., Gordon J.I., Knight R. (2009). Bacterial community variation in human body habitats across space and time. Science.

[41] Byrd A.L., Belkaid Y., Segre J.A. (2018). The human skin microbiome. Nat. Rev. Microbiol..

[42] Prescott S.L., Larcombe D.L., Logan A.C., West C., Burks W., Caraballo L., Levin M., Etten E.V., Horwitz P., Kozyrskyj A. (2017). The skin microbiome: Impact of modern environments on skin ecology, barrier integrity, and systemic immune programming. World Allergy Organ. J..

[43] Cogen A.L., Nizet V., Gallo R.L. (2008). Skin microbiota: A source of disease or defence?. Br. J. Dermatol..

[44] Flores G.E., Bates S.T., Knights D., Lauber C.L., Stombaugh J., Knight R., Fierer N. (2011). Microbial biogeography of public restroom surfaces. PLoS ONE.

[45] Cheng H., Chen J., Yu H., Sun B., Zhou J., Wu G. (2025). Environmental factors and antimicrobial efficacy: The impact of temperature and humidity on material surfaces. Microbiol. Spectr..

[46] Lopez G.U., Gerba C.P., Tamimi A.H., Kitajima M., Maxwell S.L., Rose J.B. (2013). Transfer efficiency of bacteria and viruses from porous and nonporous fomites to fingers under different relative humidity conditions. Appl. Environ. Microbiol..

[47] Rusin P., Maxwell S., Gerba C. (2002). Comparative surface-to-hand and fingertip-to-mouth transfer efficiency of gram-positive bacteria, gram-negative bacteria, and phage. J. Appl. Microbiol..

[48] Dancer S.J. (2009). The role of environmental cleaning in the control of hospital-acquired infection. J. Hosp. Infect..

[49] Amann R.I., Ludwig W., Schleifer K.-H. (1995). Phylogenetic identification and in situ detection of individual microbial cells without cultivation. Microbiol. Rev..

[50] Janda J.M., Abbott S.L. (2007). 16S rRNA gene sequencing for bacterial identification in the diagnostic laboratory: Pluses, perils, and pitfalls. J. Clin. Microbiol..

